# An expanded auxin-inducible degron toolkit for *Caenorhabditis elegans*

**DOI:** 10.1093/genetics/iyab006

**Published:** 2021-01-20

**Authors:** Guinevere E Ashley, Tam Duong, Max T Levenson, Michael A Q Martinez, Londen C Johnson, Jonathan D Hibshman, Hannah N Saeger, Nicholas J Palmisano, Ryan Doonan, Raquel Martinez-Mendez, Brittany R Davidson, Wan Zhang, James Matthew Ragle, Taylor N Medwig-Kinney, Sydney S Sirota, Bob Goldstein, David Q Matus, Daniel J Dickinson, David J Reiner, Jordan D Ward

**Affiliations:** 1 Department of Molecular, Cell, and Developmental Biology, University of California-Santa Cruz, Santa Cruz, CA 95064, USA; 2 Center for Translational Cancer Research, Institute of Biosciences and Technology, Texas A&M Health Science Center, Texas A&M University, Houston, TX 77030, USA; 3 Department of Biochemistry and Cell Biology, Stony Brook University, Stony Brook, NY 11794, USA; 4 Department of Biology, University of North Carolina at Chapel Hill, Chapel Hill, NC 27599, USA; 5 Department of Molecular Biosciences, University of Texas at Austin, Austin, TX 78712, USA; 6 Lineberger Comprehensive Cancer Center, University of North Carolina at Chapel Hill, Chapel Hill, NC 27599, USA

**Keywords:** *C. elegans*, AID system, SapTrap, self-excising cassette, CRISPR/Cas9, Transport Inhibitor Response 1

## Abstract

The auxin-inducible degron (AID) system has emerged as a powerful tool to conditionally deplete proteins in a range of organisms and cell types. Here, we describe a toolkit to augment the use of the AID system in *Caenorhabditis elegans*. We have generated a set of single-copy, tissue-specific (germline, intestine, neuron, muscle, pharynx, hypodermis, seam cell, anchor cell) and pan-somatic TIR1*-*expressing strains carrying a co-expressed blue fluorescent reporter to enable use of both red and green channels in experiments. These transgenes are inserted into commonly used, well-characterized genetic loci. We confirmed that our TIR1-expressing strains produce the expected depletion phenotype for several nuclear and cytoplasmic AID-tagged endogenous substrates. We have also constructed a set of plasmids for constructing repair templates to generate fluorescent protein::AID fusions through CRISPR/Cas9-mediated genome editing. These plasmids are compatible with commonly used genome editing approaches in the *C. elegans* community (Gibson or SapTrap assembly of plasmid repair templates or PCR-derived linear repair templates). Together these reagents will complement existing TIR1 strains and facilitate rapid and high-throughput fluorescent protein::AID tagging of genes. This battery of new TIR1-expressing strains and modular, efficient cloning vectors serves as a platform for straightforward assembly of CRISPR/Cas9 repair templates for conditional protein depletion.

## Introduction

The AID system has allowed rapid, conditional, and tissue-specific depletion of tagged proteins in a wide range of organisms and cell types (Nishimura *et al.* 2009; Holland *et al.* 2012; Zhang *et al.* 2015; Natsume *et al.* 2016; Trost *et al.* 2016; Brown *et al.* 2017; Chen *et al.* 2018; Daniel *et al.* 2018; Camlin and Evans 2019). Since its introduction to *C. elegans* (Zhang *et al.* 2015), it has been promptly adopted by the community. This system has allowed for rapid depletion of proteins in tissues that are refractory to RNA interference approaches, such as the germline (Pelisch *et al.* 2017; Shen *et al.* 2018; Zhang *et al.* 2018b), vulval precursor cells (Matus *et al.* 2014), and neurons (Liu *et al.* 2017; Patel and Hobert 2017; Serrano-Saiz *et al.* 2018). The system is also powerful for studying rapid developmental events such as molting (Zhang *et al.* 2015; Joseph *et al.* 2020), organogenesis (Martinez *et al.* 2020), developmental timing (Azzi *et al.* 2020), meiosis (Zhang *et al.* 2015), and spermatogenesis (Ragle *et al.* 2020). Improvements to the auxin ligand have enhanced protein degradation in the embryo (Negishi *et al.* 2019) and removed the need for ethanol solubilization, allowing the auxin derivative to be dissolved in any aqueous buffer (Martinez *et al.* 2020). This water-soluble auxin was shown to be compatible with microfluidic devices, allowing the coupling of long-term imaging and targeted protein depletion (Martinez *et al.* 2020). Auxin-mediated depletion of a spermatogenesis regulator has been developed to conditionally sterilize animals, a valuable approach for the *C. elegans* aging field (Kasimatis *et al.* 2018).

**Figure 1:**
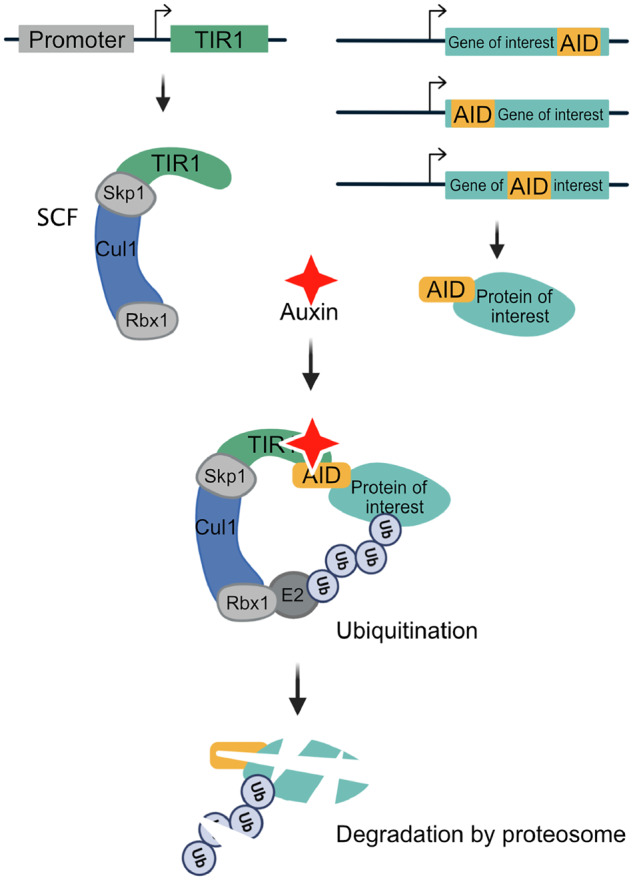
Schematic of the auxin-inducible degron (AID) system. The plant F-box protein TIR1 is expressed using a promoter of interest with a desired spatiotemporal expression pattern. TIR1 interacts with endogenous Skp1 and Cul1 proteins to form an SCF E3 ubiquitin ligase complex. An auxin-inducible degron sequence (AID) is fused to a protein of interest. In the presence of the plant hormone auxin, TIR1 recognizes and binds the AID sequence, leading to ubiquitination and subsequent degradation of the AID-tagged protein. We use a minimal, 44 amino acid degron sequence (AID*), but a full-length 229 amino acid AID tag or a 68 amino acid mini-AID (mAID) are used in other systems. In *C. elegans*, the system is frequently used with single-copy TIR1 transgenes inserted into neutral loci, and AID* knock-ins into genes of interest, though extrachromosomal arrays can also be used. Created with BioRender.com.

The system is comprised of two components. First, the plant F-box protein Transport Inhibitor Response 1 (TIR1) is expressed under the control of a promoter with a defined expression pattern (Figure 1). TIR1 can then interact with endogenous Skp1 and Cul1 proteins to form a functional SCF E3 ubiquitin ligase complex (Figure 1). Second, an auxin-inducible degron (AID) sequence from the IAA17 protein is fused to a protein of interest (Figure 1) (Nishimura *et al.* 2009; Natsume and Kanemaki 2017). Addition of the plant hormone, auxin, promotes TIR1 binding to the degron, leading to the ubiquitination and subsequent proteasome-mediated degradation of the degron-tagged protein (Figure 1). Although the full-length IAA17 sequence is 229 amino acids, minimal AID tags of 44 amino acids (AID*) and 68 amino acids (mAID) have been developed (Morawska and Ulrich 2013; Li *et al.* 2019). In *C. elegans*, the *Arabidopsis thaliana* TIR1, AID*, and mAID sequences are used (Zhang *et al.* 2015; Negishi *et al.* 2019), as this plant grows at a temperature range more similar to *C. elegans*, whereas rice (*Oryza sativa*)-derived sequences are used in other systems (Nishimura *et al.* 2009; Natsume *et al.* 2016; Natsume and Kanemaki 2017). The SCF ligase in *C. elegans* with which TIR1 interacts is thought to be comprised of SKR-1/2, CUL-1, and RBX-1 (Martinez *et al.* 2020).

Here, we describe a new set of strains and reagents for *C. elegans* that complement the tools originally described by Zhang *et al.* (2015). To enable both targeted protein degradation and imaging-based measurement of depletion, we have generated a set of strains that express single-copy, tissue-specific TIR1 marked with a reporter of activity (TIR1::F2A::mTagBFP2::AID*::NLS). Nuclear-localized mTagBFP2::AID* (hereafter referred to simply as “BFP”) serves as a reporter for TIR1 expression. TIR1 should degrade BFP in the presence of auxin, providing a built-in read-out of TIR1 activity. For many of the tissue-specific promoters driving this construct, we have created strains with insertions into well-characterized, neutral target sites on both chromosomes I and II (Frøkjaer-Jensen *et al.* 2008; Frøkjær-Jensen *et al.* 2012), to facilitate crossing schemes. These strains expand experimental possibilities with green and red fluorescent proteins (FPs) of interest. We have also generated constructs to introduce FP::AID* tags into genes of interest using conventional genome editing approaches in *C. elegans*.

## Methods

### Molecular biology

In the below constructs, flexible linker sequences ranging from 5-9 glycine/serine residues are used to separate cassettes within constructs (*i.e*., AID*, 3xFLAG, 3xMyc, TEV protease recognition sites, etc.). Unless otherwise specified, pJW plasmids (Ward lab) were generated by Gibson cloning using an in-house-made master mix, as described (Gibson *et al.* 2009). For two-fragment Gibson cloning, 0.63 µl of each DNA fragment was mixed with 3.75 µl of the Gibson master mix and incubated for 1-4 hours at 50°C. Longer reaction times were used for inefficient assemblies. Reactions were then transformed as described in the supplemental methods or stored at -20°C. Detailed methods describing construct generation are provided in supplemental methods (Supplementary File S2). Oligos used to construct plasmids are listed in Supplementary Table S1. All plasmids used are listed in Supplementary Table S2. Primer design information for designing homology arms for Gibson and SapTrap cloning is provided in Supplementary File S3. Supplementary File S4 contains sequence files for all plasmids generated for this study.

### Caenorhabditis elegans

*Caenorhabditis elegans* strains were cultured as originally described (Brenner 1974). All strains used in this study are listed in Supplementary Table S3. The majority of genome editing was performed in N2 (wild type), EG9615 *oxSi1091[mex-5p::Cas9(smu-2 introns) unc-119+] II; unc-119(ed3) III*, or EG9882 *F53A2.9(oxTi1127[mex-5p::Cas9::tbb-2 3'UTR, Phsp-16.41::Cre::tbb-2 3'UTR, Pmyo-2::nls-CyOFP::let-858 3'UTR + lox2272]) III* animals (Supplementary Table S3). EG9615 and EG9882 (unpublished) stably express Cas9 in the germline and are gifts from Dr. Matthew Schwartz and Dr. Erik Jorgensen. TIR1 was isolated by outcrossing and loss of the *Cas9* allele was confirmed by PCR or loss of *myo-2::NLS::OFP* expression. The *mex-5p, myo-2p* and *cdh-3p* strains were generated in specialized genetic backgrounds and then the TIR1 transgene was isolated by outcrossing. The loss of other alleles in the background was confirmed by PCR genotyping. Details are provided in Supplemental File 2 and Supplementary Table S4. Specific details for the TIR1 strains (injection strain, Cas9 and sgRNA source, number of times outcrossed to an N2 background) are provided in Supplementary Table S4. We note that we are reporting the final, SEC-excised strains in this table. We have made the precursor strains containing the SEC available to the CGC for our *mex-5*p and *eft-3p* strains: JDW220 *wrdSi10[mex-5p::TIR1::F2A::BFP::tbb-2 3'UTR+SEC]* I:-5.32, JDW222 *wrdSi8[mex-5p::TIR1::F2A::BFP::tbb-2 3'UTR + SEC]* II:-0.77, and JDW224 *wrdSi22[eft-3p::TIR1::F2A: BFP::tbb-2 3'UTR+SEC]* I:-5.32. Following SEC-excision, chromosome I knock-ins can be PCR genotyped using oligos 2835 + 2836 + 3415 (Supplementary Table S1). A wild-type locus produces a 623 bp band and the TIR1 knock-in produces an 881 bp band. Chromosome II knock-ins can be similarly genotyped post-SEC excision by PCR with oligos 273 + 274 + 3415 (Supplementary Table S1). A wild-type locus produces a 684 bp band and a TIR1 knock-in produces an 881 bp band.

### CRISPR/Cas9-based genome editing

All TIR1 strains were generated through SEC selection-based genome editing as previously described (Dickinson *et al.* 2015). Single-copy transgenes were inserted into the chromosome I and II loci where the ttTi4348 and ttTi5605 transposons are inserted for MosSCI (Frøkjaer-Jensen *et al.* 2008; Frøkjær-Jensen *et al.* 2012), respectively. The genetic map positions for these insertions are provided in the genotype information. In Supplementary Table S5, we detail the genetic background in which injections were performed, which Cas9 and sgRNA plasmids were used, and how many times the strains were outcrossed against an N2 background. Repair templates were used at 10 ng/µl and Cas9+sgRNA or sgRNA plasmids were used at 50 ng/µl.

### Auxin treatment

The Dickinson and Reiner labs routinely use 1 mM IAA, whereas the Matus and Goldstein lab routinely use 1 mM K-NAA for experiments. The Ward lab routinely uses 4 mM IAA as a stronger NHR-25 depletion phenotype (embryonic lethality) is observed at 4 mM IAA, but not at 1 mM IAA (Zhang *et al.* 2015). In addition, a sterility phenotype produced by depleting NHR-23 in the germline is more penetrant and reproducible on 4 mM IAA compared to 1 mM IAA (unpublished data). Therefore, the western blots (Figures 3B and 4A) and phenotypic assays (Tables 2 and 3; Supplementary Tables S5 and S6) were performed using 4 mM IAA or K-NAA to provide the strongest possible depletion. For DQM623 imaging (Figure 7), we also used a higher concentration of auxin (4 mM K-NAA), as we could not detect BFP reporter expression from the *bmd176[cdh-3pTIR1::F2A::mTagBFP2::AID::NLS]* transgene and wanted to design the experiment to achieve the strongest possible depletion of NHR-25. Controls for experiments using IAA are NGM plates with an equivalent concentration of ethanol vehicle. Controls for experiments using K-NAA are NGM plates.

### BFP::AID*::NLS reporter depletion experiments

**Figure 2:**
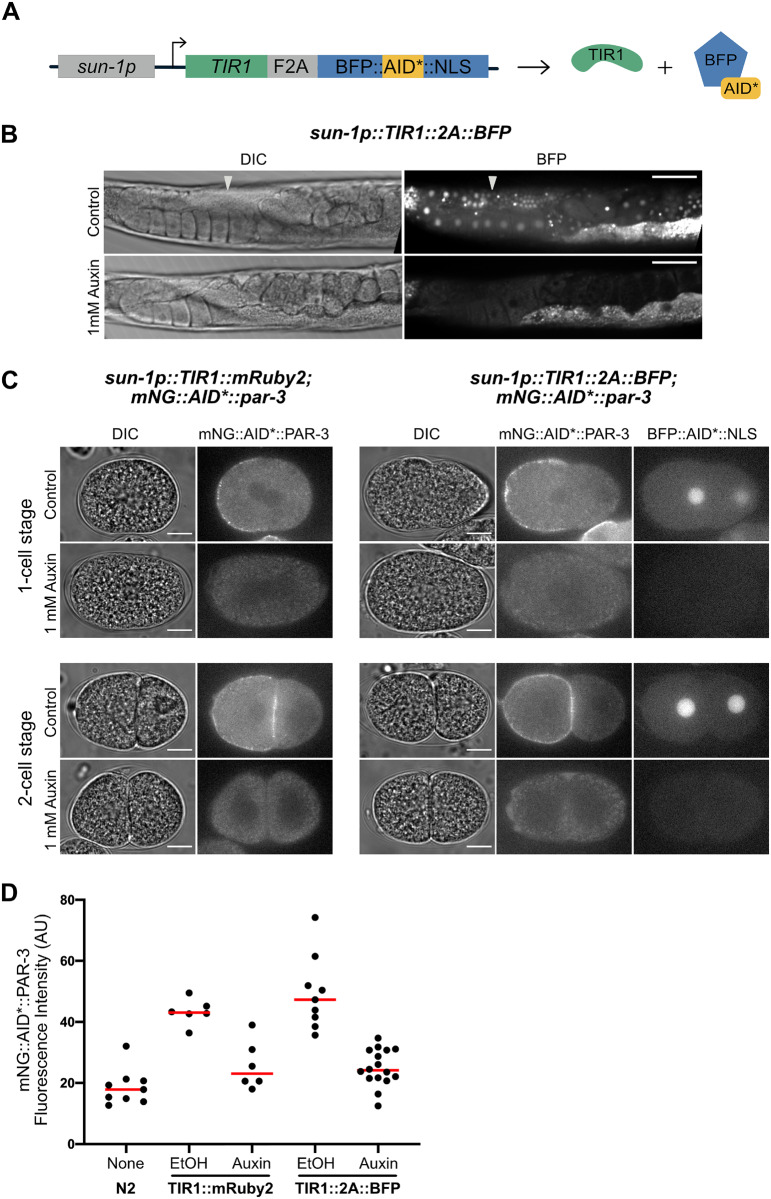
A new TIR1 expression system allows assessment of TIR1 expression and activity. (A) The new TIR1 expression construct contains a TIR1::F2A::BFP::AID*::NLS transgene cassette. An F2A skip sequence results in expression of two separate protein products: 1) TIR1, which will interact with endogenous SCF proteins to produce an E3 ubiquitin ligase complex and can only bind the AID sequence in the presence of auxin; and 2) an AID*-tagged BFP protein with a c-Myc nuclear localization signal (NLS) that functions as a readout for TIR1 expression and internal control for TIR1 activity. The use of BFP as a reporter makes this construct compatible with simultaneous green and red FP imaging. Created with BioRender.com. (B) Adult animals expressing sun1p::TIR1::F2A::BFP::AID*::NLS. A control animal expresses AID*-tagged BFP in the nuclei of germline and embryonic cells (white arrows). When animals are exposed to 1 mM auxin, BFP expression is undetectable. BFP channel and DIC images are provided for each condition. Note that the fluorescence signal at the lower right-hand side of each BFP image is due to intestinal autofluorescence. Scale bars represent 50 µm. (C) Images of embryos harboring endogenously-tagged mNG::AID*::PAR-3 and expressing either *sun-1p::TIR1::mRuby2* (Zhang et al. 2015) or sun-1p::TIR1::F2A::BFP::AID*::NLS (this study). Both TIR1 transgenes were able to deplete mNG::AID*::PAR-3 to background levels in the presence of auxin and produced a symmetric first division as expected for loss of PAR-3 function. D) Quantification of whole-embryo mNG::AID*::PAR-3 fluorescence intensity for the indicated conditions. Wild-type (N2) embryos were measured to account for autofluorescent background. Data points represent the fluorescence intensity from individual embryos; at least six embryos were imaged for each condition. The horizontal red bar depicts the mean for each condition.

For Figure 2B, LP630 cpIs103[sun-1p::TIR1-C1::F2A::mTagBFP2-C1::AID*::NLS + SEC II:-0.77] II L4 larvae were placed on standard NGM plates containing 1 mM Auxin or 4% ethanol (control) and kept at 22°C overnight. Young adults were the mounted on 3% agar pads containing 10 mM sodium azide as a paralytic. Images were acquired on a Nikon TiE microscope equipped with a 405 nm diode laser for fluorescence excitation; a 20x air objective and 1.2x tube lens (total magification of 24x); a CSU-X1 spinning disk head; and a Hamamatsu ImageEM EM-CCD camera. Acquisition was controlled by MetaMorph software, and images were prepared for display by cropping, rotating and adjusting brightness and contrast using ImageJ. No other image manipulations were performed.

For DV3799 reSi1[col-10p::TIR1::F2A::mTagBFP2::AID*::NLS::tbb-2 3'UTR I:-5.32], DV3801 reSi3[unc-54P::TIR1::F2A::mTagBFP2::AID*::NLS::tbb-2 3'UTR I:-5.32], DV3803 reSi5[ges-1p::TIR1::F2A::mTagBFP2::AID*::NLS::tbb-2 3'UTR I:-5.32], and DV3805 reSi7[rgef-1p::TIR1::F2A::mTagBFP2::AID*::NLS::tbb-2 3'UTR I:-5.32] (Figure 6), we made an auxin (IAA) (Alpha Aesar, #A10556) stock solution (400 mM in ethanol) that was stored at 4˚C for up to 1 month. A 16 mM auxin working solution was then prepared freshly by diluting 1:25 in filtered water with 4% ethanol final concentration. 500 µL of 16 mM auxin was added to 60 mm NGM plates for a final concentration of 1 mM, with 4% ethanol as vehicle control (plates contain approximately 8 ml of agar). Plates were then seeded with OP50. Larvae were grown on NGM plates to the desired stage, then shifted onto auxin or vehicle plates for 3 hours before imaging. Animals were anesthetized with 5 mM tetramisole and images were acquired on a Nikon A1si Confocal Laser Microscope using a Plan-Apochromat 40x/1.4 DIC objective and DS-Fi2 camera. Images were analyzed using NIS Elements Advanced Research, Version 4.40 software (Nikon).

For JDW221 wrdSi18[mex-5p::TIR1::F2A::mTagBFP2::AID*::NLS::tbb-2 3'UTR I:-5.32], JDW225 wrdSi23[eft-3p::TIR1::F2A::mTagBFP2::AID*::NLS::tbb-2 3'UTR I:-5.32], JDW229 wrdSi47[dpy-7p::TIR1::F2A::mTagBFP2::AID*::NLS::tbb-2 3'UTR I:-5.32] (Supplementary Figure S4), 1-hour auxin treatments were performed. Animals were synchronized at the L1 larval stage by sodium hypochlorite treatment and moved to nematode growth media (NGM) plates seeded with *E. coli* OP50. At the young adult stage, JDW221 worms were either kept on OP50-seeded NGM plates (control) or moved to plates treated with 1 mM K-NAA (Phyto-Technology Laboratories, N610) (Martinez and Matus 2020). At the mid-L3 larval stage, JDW225 and JDW229 worms were either kept on OP50-seeded NGM plates or moved to plates treated with 1 mM K-NAA. OP50-seeded NGM plates containing K-NAA were prepared as described (Martinez and Matus 2020). Images were acquired on a custom-built upright spinning-disk confocal microscope consisting of a Zeiss Axio Imager.A2 modified with a Borealis-modified Yokogawa CSU-10 confocal scanner unit with 405 nm lasers and 488 nm lasers and equipped with a Hamamatsu Orca EM-CCD camera. Images shown for JDW221 (pachytene region) were acquired using a Plan-Apochromat 40x/1.4 DIC objective. Images shown for JDW225 (uterine and vulval tissues) and JDW229 (hypodermal cells) were acquired using a Plan-Apochromat 100x/1.4 DIC objective. MetaMorph software (version: 7.8.12.0) was used to automate image acquisition. Worms were anesthetized on 5% agarose pads containing 7 mM NaN_3_ and secured with a coverslip. Acquired images were processed through Fiji software (version: 2.0.0- rc-69/1.52p).

For LP869 cpSi171[vha-8p::TIR1::F2A::mTagBFP2::AID*::NLS::tbb-2 3'UTR I:-5.32], LP870 cpSi172[myo-2p::TIR1::F2A::mTagBFP2::AID*::NLS::tbb-2 3'UTR I:-5.32], and LP871 cpSi174[myo-3p::TIR1::F2A::mTagBFP2::AID*::NLS::tbb-2 3'UTR I:-5.32]Supplementary Figure S4), mixed stage animals were transferred to 1 mM K-NAA NGM plates for 24 hours. Images were then taken using the 60x objective on a Nikon TiE stand with CSU-X1 spinning disk head (Yokogawa), 447 nm, 514 nm, and 561 nm solid state lasers, ImagEM EMCCD camera (Hamamatsu). Worms were anesthetized and images were processed as described above.

### AID*-tagged substrate depletion experiments

To examine depletion of mNG::AID*::PAR-3 in embryos (Figure 2), young adult worms were treated with either 1 mM IAA or 0.25% EtOH in standard liquid culture conditions (Stiernagle 2006) for 1 hour. Then, embryos were dissected on polylysine-coated coverslips and mounted in egg buffer with 22.8 µm beads as spacers. Confocal images were captured on a Nikon Ti2 microscope equipped with 405 nm and 488 nm illumination lasers; a 60x, 1.4 NA oil immersion objective; a Visitech VT-iSIM scan head; and a Photometrics PrimeBSI camera controlled by µManager software. The total amount of mNG::AID*::PAR-3 present in each embryo was measured in FIJI by drawing a box around each embryo, measuring integrated pixel intensity, and then converting to a fluorescence intensity by subtracting off-embryo background. Results were plotted using Graphpad Prism software.

To monitor depletion of GFP::AID in the vulval precursor cells (Figure 4, B and C) CA1202 ieSi57 *[eft-3p::TIR1::mRuby::unc-54 3'UTR + Cbr-unc-119(+)] II; ieSi58 [eft-3p::degron::GFP::unc-54 3'UTR + Cbr-unc-119(+)] IV* and JDW185 wrdSi54[eft-3p: TIR1: F2A: mTagBFP2: tbb-2 3'UTR, I:-5.32]; unc-119(ed3) III; ieSi58 [eft-3p::AID*::GFP::unc-54 3'UTR + Cbr-unc-119(+)] IV animals were grown on NGM plates until early L3. A subset of animals were imaged at timepoint “0 min,” and the remainder were shifted onto 1 mM K-NAA and imaged at the indicated timepoints. Images were acquired using a Plan-Apochromat 100x/1.4 DIC objective on a custom-built upright spinning-disk confocal microscope. This microscope consisted of a Zeiss Axio Imager.A2 modified with a Borealis-modified Yokogawa CSU-10 confocal scanner unit with 405 nm lasers and 488 nm lasers and equipped with a Hamamatsu Orca EM-CCD camera. MetaMorph software (version: 7.8.12.0) was used to automate image acquisition. Worms were anesthetized on 5% agarose pads containing 7 mM NaN_3_ and secured with a coverslip. Acquired images were processed through Fiji software (version: 2.0.0- rc-69/1.52p). GFP was quantified as described in Martinez et al. (2020). Measurements of total fluorescence intensity in the nucleus was measured, and fluorescence intensity immediately outside the circumference of the nucleus was also measured to obtain the cytoplasmic value. The ratio of these intensities is plotted in Supplementary Figure S3. For Figure 4C, only the nuclear intensity was plotted.

To monitor depletion of nuclear NHR-25::GFP::AID* in the vulval precursor cells and seam cells using *eft-3p::TIR1* transgenes (Figure 5), JDW71 ieSi57 [eft-3p::TIR1::mRuby2::unc-54 3’UTR, cb-unc-119(+)] II; unc-119(ed3) III; nhr-25(wrd18[nhr-25::GFP::AID*:3xFLAG]) X and JDW187 wrdSi23[eft-3p::TIR1: F2A: mTagBFP2:: AID*::NLS: tbb-2 3'UTR, I:-5.32]; wrd52[nhr-25::GFP::AID*::3xFLAG] X animals were grown to L3 on NGM plates. Animals were auxin treated, and they were imaged and analyzed as described above for AID*::GFP depletion experiments. Experiments measuring NHR-25::GFP::AID* depletion using DQM623 bmd176[cdh-3pTIR1::F2A::mTagBFP2::AID*::NLS] II; nhr-25(wrd10[nhr-25::GFP^AID*:3xFLAG]) X were similarly performed (Figure 7), except animals were imaged one hour after exposure to control or 4 mM K-NAA plates.

### Western blot analysis

**Figure 3:**
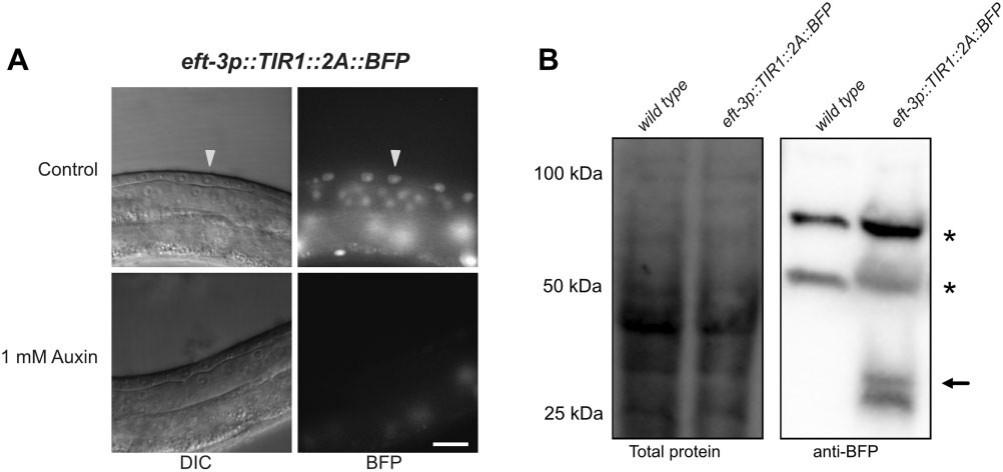
The F2A ribosome skip sequence functions efficiently in an eft-3p::TIR1::F2A::BFP::AID*::NLS transgene. (A) L3 larvae expressing eft-3p:: TIR1::F2A::BFP::AID*::NLS. A control animal expresses AID*-tagged BFP in the nuclei of vulval precursor cells (VPCs; white arrows). BFP expression is undetectable in animals grown on 1 mM K-NAA—a water-soluble, synthetic auxin—for 1 hour before imaging. Scale bars represent 15 µm (*eft-3p*). (B) Western blot detecting *BFP::AID::NLS*. Stain-free (Bio-Rad) analysis of total protein on the blot is provided as a loading control (left). Marker size (in kilodaltons) is provided. Anti-BFP blot showing background bands (marked with *) and a doublet consistent with the predicted size of BFP::AID*::NLS (black arrow) at approximately 34.5 kDa, and a smaller band below, likely a BFP degradation product.

**Figure 4:**
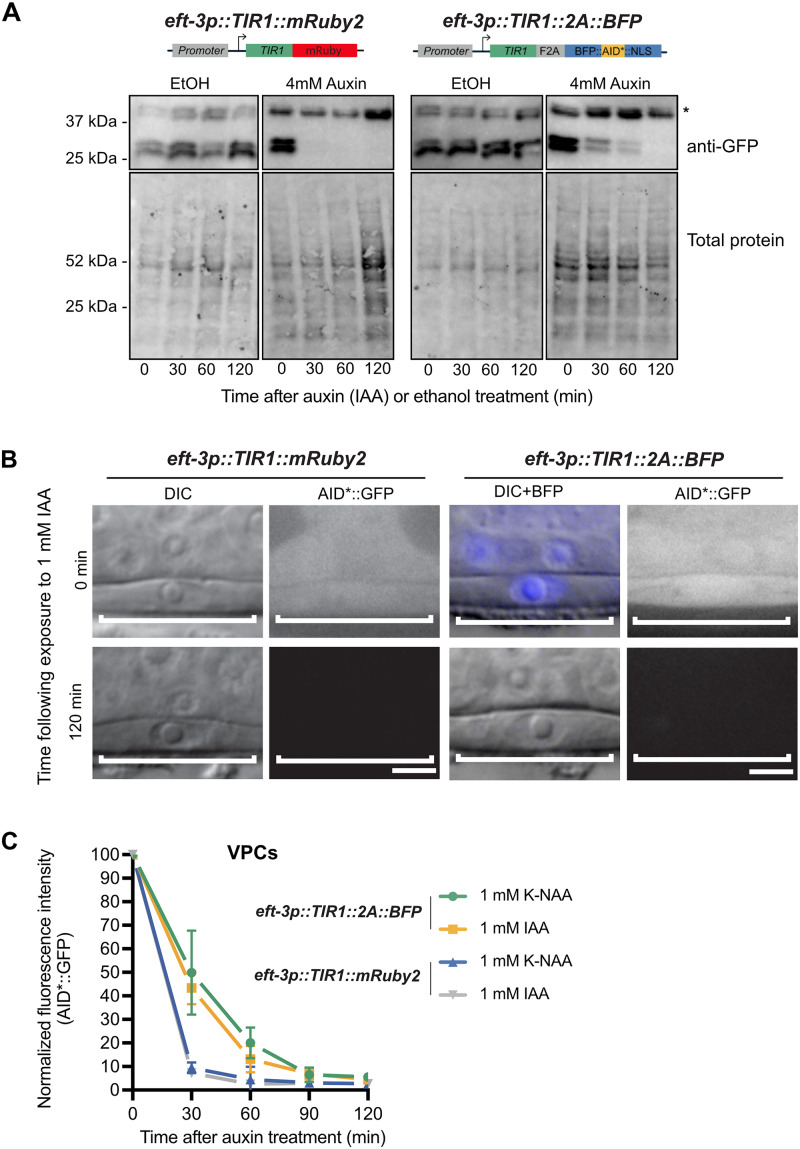
eft-3p::TIR1::F2A::BFP::AID*::NLS depletes AID*::GFP to the same extent as *eft-3p::TIR1::mRuby2* but produces a slower rate of degradation. (A) Western blots detecting AID*::GFP after exposure to IAA or EtOH (control). An AID*::GFP reporter strain was crossed to either *eft-3p::TIR1::mRuby2* (left) or eft-3p::TIR1::F2A::BFP::AID*::NLS (right) and then exposed to 4 mM IAA or EtOH for 0 min, 30 min, 60 min, or 120 min. Anti-GFP blots (top) showing background band (marked with *) and a doublet at approximately 27 kDa, consistent with the predicted size of GFP. Created with BioRender.com. (B) Representative images of AID*::GFP depletion in animals carrying either *eft-3p::TIR1::mRuby2* or eft-3p::TIR1::F2A::BFP::AID*::NLS. For animals expressing eft-3p::TIR1::F2A::BFP::AID*::NLS, an overlay of DIC and BFP images is provided. DIC and corresponding GFP images of VPCs (brackets) from L3 larvae at the P6.p 1-cell stage. Animals were treated with 1 mM IAA for the specified time and then imaged to visualize loss of AID*::GFP. Representative images from additional timepoints can be found in Supplementary Figure S2. Scale bars represent 5 µm. (C) AID*::GFP degradation kinetics. Rates of degradation were determined by quantifying AID*::GFP levels in VPCs of animals as described above. Animals were exposed to 1 mM K-NAA or IAA from 0 to 120 minutes at intervals of 30 min. The graph depicts the mean normalized fluorescent intensity from 10 or more animals from a single experimental replicate. Error bars indicate standard deviation.

For all western blots, animals were synchronized by sodium hypochlorite treatment, moved to nematode growth media (NGM) plates seeded with *E. coli* OP50, and grown at 20°C for ∼40 hours. L4 animals were collected untreated or exposed to 4 mM auxin (IAA) or ethanol for the amount of time specified prior to collection in M9 buffer. Animals were washed several times, pelleted, and freeze cracked in liquid nitrogen 2X. To each worm sample was added house-made 4x Laemmli Sample Buffer, followed by 10 minutes boiling at 95°C and centrifugation. The resulting supernatants were used for western blot analysis. The total proteins were denatured for 10 min at 95°C. Equal amounts of protein samples were loaded and separated by precast 4-20% Mini-Protean TGX Stain Free Gels (Bio-Rad) then transferred to polyvinylidene difluoride membranes. The primary antibodies used were rabbit anti-GFP antibody (Abcam, #ab290) (Figure 4 and Supplementary Figure S1), and rabbit anti-tRFP (Evrogen, #AB233) (Figure 3) both diluted at 1:1000. The secondary antibody used was goat anti-Rabbit-HRP (Kindle Biosciences LLC, #R1006) diluted at 1:1000 in all cases. Images were captured with a Bio-Rad ChemiDoc imaging system.

### Phenotypic analysis

For all strains containing nhr-23::GFP::AID* and nhr-25::GFP::AID* (Table 2 and Supplementary Table S5), experiments were performed as previously described (Zhang *et al.* 2015). Briefly, adult hermaphrodites were picked onto MYOB plates seeded with OP50 and containing 4 mM IAA, 4 mM K-NAA, or EtOH (control). Adults were permitted to lay eggs for 4 hours at 25°C prior to being removed and the resulting progeny were kept at 25°C and scored daily for larval arrest and gonadal abnormalities for 4 days. For strains containing daf-15::mNG::AID* (Table 3 and Supplementary Table S6), animals were synchronized at L1 by sodium hypochlorite treatment and released onto MYOB plates seeded with OP50 and containing either 4 mM auxin (IAA or K-NAA), or EtOH (control). Animals were grown at 20°C for 4 days and scored for larval arrest. Larval arrest stage (L2 *vs* L3) was determined by animal size and confirmed by imaging a subset of animals by DIC microscopy and evaluating gonadal development.

### Statistical analysis

Statistical significance was determined using a two-tailed unpaired Student’s t-test. *P *<* *0.05 was considered statistically significant. **** indicates *P *<* *0.0001.

### Data availability

Strains in Figure 6A and Supplementary Table S4 are available through the *Caenorhabditis* Genetics Center with the exception of DQM526 which can be obtained upon request from Prof. David Matus. Other strains and plasmids can be requested directly from the authors. The data that support the findings of this study are available upon reasonable request. The indicated plasmids in Supplementary Table S2 are available through Addgene. Supplemental files are available at figshare: https://doi.org/10.25386/genetics.12280994.

## Results

### A new TIR1 transgene with a built-in BFP::AID*::NLS reporter

The initial description of the AID system in *C. elegans* used an mRuby2 fusion to monitor the expression of TIR1 (Zhang *et al.* 2015). This feature was useful to monitor TIR1 localization and expression level in comparison to depletion of GFP::AID* tagged proteins. One limitation is that the mRuby2 fusion interferes with the imaging of factors tagged with red FPs. Blue fluorescent proteins offer an appealing alternative as reporters for TIR1 expression, as their emission spectra do not significantly overlap with commonly used green and red FPs (Lambert 2019). To report TIR1 expression and activity, we placed an F2A::BFP::AID*::NLS reporter downstream of the TIR1 transgene and put the transgene under the control of a *sun-1* promoter, which drives germline and embryo expression (Figure 2A). We used an F2A ribosome skip sequence to allow separate TIR1 and BFP::AID*::NLS proteins to be produced from the mRNA transcript (Ryan and Drew 1994; Ryan *et al.* 1999; Donnelly *et al.* 2001; de Felipe *et al.* 2003; Ahier and Jarriault 2014).

Our sun-1p::TIR1::F2A::BFP::AID*::NLS transgene was introduced in single copy through CRISPR/Cas9 editing and self-excising cassette (SEC) selection into the same neutral locus where MosI transposon was inserted in ttTi4348 (Dickinson *et al.* 2015). The SEC strategy (Dickinson *et al.* 2015) first produces hygromycin resistant, rolling animals, which is useful for tracking the allele phenotypically in crosses. The loxP-flanked SEC is then excised by heat shock, producing the final strain with wild type locomotion. As expected, this *sun-1p* construct drives nuclear-localized BFP in the germline and embryos, confirming the expression of the transgene. We confirmed TIR1 activity by placing adult animals on 1 mM auxin and observing loss of BFP::AID*::NLS (Figure 2B). To test the performance of our new TIR1 transgene, we compared depletion of mNeonGreen (mNG)::AID*::PAR-3 using the sun-1p::TIR1::F2A:::BFP::AID*::NLS transgene and the original *sun-1p::TIR1::mRuby2* transgene (Zhang *et al.* 2015). In the absence of auxin, comparable PAR-3 fluorescence was observed (Figure 2, C and D). Similarly, exposure to 1 mM auxin resulted in a comparable loss of fluorescence (Figure 2D). Moreover, 100% of auxin-treated embryos showed symmetric cell division which is the expected *par-3* loss-of-function phenotype (Figure 2C). Together, these results indicate that our TIR1::F2A::BFP::AID*::NLS transgene can effectively deplete AID*-tagged PAR-3, a cytoplasmic cell polarity determinant.

We planned to insert our constructs into the sites in chromosomes I and II, respectively, where the ttTi4348 and ttTi5605 transposons are inserted for MosSCI-based genome editing (Frøkjaer-Jensen *et al.* 2008; Frøkjær-Jensen *et al.* 2012). To facilitate genome editing, we created sgRNA and Cas9+sgRNA vectors targeting these loci containing the “Flipped and extended (F + E)” sgRNA modifications that improved editing efficiency in mammalian cells and *C. elegans* (Chen *et al.* 2013; Ward 2015) (Table 1). We generated sgRNA expression vectors using the two commonly used U6 promoters in *C. elegans* as it is currently unclear whether one promoter is broadly more active (Dickinson *et al.* 2013; Friedland *et al.* 2013; Schwartz and Jorgensen 2016) (Table 1). We also generated sgRNA vectors targeting the cxTi10882 insertion site on chromosome IV to support future knock-ins into this locus (Frøkjaer-Jensen *et al.* 2008; Frøkjær-Jensen *et al.* 2012).

**Table 1:** Plasmids to support generation of new TIR1 alleles and other transgenes at standardized genetic loci through CRISPR/Cas-based genome editing

Plasmid name	Description	Purpose
pJW1838	SapTrap sgRNA (F + E) vector, *K09B11.2* U6 promoter and 3'UTR	Creating new sgRNA (F + E) plasmids)
pJW1839	SapTrap sgRNA (F + E) vector, *R07E5.16* U6 promoter	Creating new sgRNA (F + E) plasmids)
pJW1836	promoterless *SV40 NLS::mScarlet-I (dpi)::PEST::tbb-2* 3'UTR vector	Promoter reporter, test new tissue-specific promoters
pJW1841	promoterless *SV40 NLS::mScarlet-I (dpi)::tbb-2* 3'UTR vector	Promoter reporter, test new tissue-specific promoters
pJW1849	ttTi4348 site targeting sgRNA (F + E) with *K09B11.2 U6* promoter and 3'UTR	CRISPR/Cas9 editing of ttTi4348 insertion site
pJW1850	ttTi5605 site targeting sgRNA (F + E) with *K09B11.2 U6* promoter and 3'UTR	CRISPR/Cas9 editing of ttTi5605 insertion site
pJW1851	cxTi10882 site targeting sgRNA (F + E) with *K09B11.2 U6* promoter and 3'UTR	CRISPR/Cas9 editing of cxTi10882 insertion site
pJW1882	ttTi4348 site targeting sgRNA (F + E) with *R07E5.16 U6* promoter and 3'UTR	CRISPR/Cas9 editing of ttTi4348 insertion site
pJW1883	ttTi5605 site targeting sgRNA (F + E) with *R07E5.16 U6* promoter and 3'UTR	CRISPR/Cas9 editing of ttTi5605 insertion site
pJW1884	cxTi10882 site targeting sgRNA (F + E) with *R07E5.16 U6* promoter and 3'UTR	CRISPR/Cas9 editing of cxTi10882 insertion site
pJW1947	*pes-10Δ minimal promoter*::*SV40 NLS::mScarlet-I (dpi)::tbb-2 3'UTR* vector	Promoter reporter, test new tissue-specific enhancers
pJW1948	*pes-10Δ minimal promoter*::*SV40 NLS::mScarlet-I (dpi)::PEST::tbb-2 3'UTR* vector	Promoter reporter, test new tissue-specific enhancers
pTD77	*eft-3p::Cas9, R07E5.16 U6::sgRNA* targeting LGI site where ttTi4348 Mos is inserted	CRISPR/Cas9 editing of ttTi4348 insertion site
pTD78	*eft-3p::Cas9, R07E5.16 U6::sgRNA* targeting LGII site where ttTi5605 Mos is inserted	CRISPR/Cas9 editing of ttTi5605 insertion site

### The F2A ribosome skip sequence functions efficiently in an eft-3p::TIR1::F2A::BFP::AID*::NLS transgene

We tested the efficiency of the F2A peptide in our new TIR1 transgenes, as inefficient processing could create a TIR1::F2A::BFP::AID*::NLS fusion protein that could be nuclear localized and degraded, impairing performance of the system. The expression of BFP in our *sun-1*p strain was quite dim and we were unable to detect it via western blot. We therefore generated a new TIR1::F2A::BFP::AID*::NLS construct driven by a strong ubiquitous promoter (*eft-3p*) and inserted this transgene in the same site. We observed somatic BFP which was lost upon auxin exposure, indicating that our TIR1 transgene was functional (Figure 3A). We performed anti-BFP western blots on this strain (Figure 3B). We observed a doublet in the *eft-3p*::TIR1::F2A::BFP::AID*::NLS strain which was not present in N2 control animals. The upper band was consistent with the predicted size of the BFP reporter (34.5 kDa). The lower band is most likely a degradation product, as tRFP-derived proteins such as BFP are known to fragment in western blots (Kovacs *et al.* 2012; Breunig *et al.* 2015; Katoh *et al.* 2016). A similar doublet was also reported in the product literature for the antibody we used. We never observed a 101.5 kDa band, which is the predicted size for a TIR1::F2A::BFP::AID*::NLS fusion protein. Together, these data suggest that the F2A peptide is being effectively processed to produce a BFP::AID*::NLS reporter which marks TIR1 expression.

### eft-3p::TIR1::F2A::BFP::AID*::NLS is functionally equivalent to *eft-3p::TIR1::mRuby2*, but shows slower kinetics for degradation of substrates

To test the efficiency of our new eft-3p::TIR1::F2A::BFP::AID*::NLS transgene, we crossed it to an eft-3p::AID*::GFP reporter and compared AID*::GFP depletion level and rate between our new TIR1 strain and the original *eft-3p::TIR1::mRuby2* strain (Zhang *et al.* 2015). TIR1::mRuby2 strongly depleted the AID*::GFP reporter within 30 minutes of exposure to the natural auxin indole-3-acetic acid (IAA) used by Zhang *et al.* (2015). In contrast, TIR1::F2A::BFP::AID*::NLS took 120 minutes to completely deplete the AID*::GFP reporter after exposure to IAA. (Figure 4A). Single-cell imaging of the AID*::GFP reporter in vulval precursor cells (VPCs) (Figure 4B and Supplementary Figure S2) supported the western blot data (Figure 4A). We also compared the performance of IAA to K-NAA, a water-soluble synthetic auxin recently described for use in *C. elegans* (Martinez *et al.* 2020). The reporter was strongly depleted 30 minutes after exposure to both IAA and K-NAA in the TIR1::mRuby2 strain, but took 90 minutes for equivalent depletion in the TIR1::F2A::BFP::AID*::NLS strain (Figure 4C and Supplementary Figure S2). We also compared the ratio of nuclear to cytoplasmic AID*::GFP to test whether there were differences in sub-cellular depletion rates. We did not observe differences in the nuclear/cytoplasmic ratio, suggesting that both TIR1::mRuby2 and TIR1:: F2A::BFP::AID*::NLS are equally effective at depleting nuclear and cytoplasmic AID*::GFP (Supplementary Figure S3). IAA and K-NAA performed identically in these experiments (Supplementary Figure S3).

**Figure 5:**
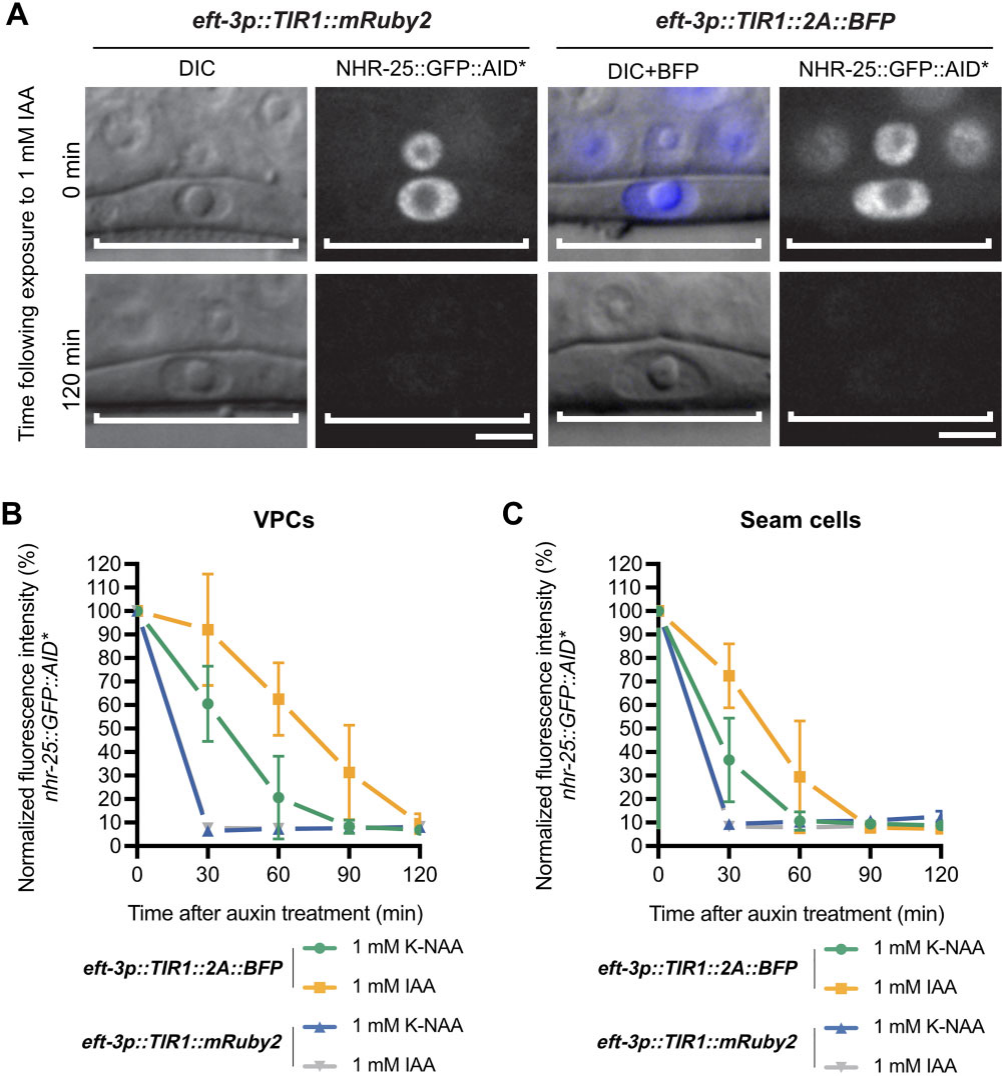
eft-3p::TIR1::F2A::BFP::AID*::NLS depletes NHR-25::GFP::AID* to the same extent as *eft-3p::TIR1::mRuby2* but also shows a slower degradation rate. (A) Representative images of NHR-25::GFP::AID* depletion in VPCs of animals expressing either *eft-3p::TIR1::mRuby2* or eft-3p::TIR1::F2A::BFP::AID*::NLS. For animals expressing eft-3p::TIR1::F2A::BFP::AID*::NLS, an overlay of DIC and BFP images were used to show BFP internal control expression in VPCs. DIC and corresponding GFP images of VPCs (brackets) from L3 larvae at the P6.p 1-cell stage. Animals were treated with 1 mM IAA for the specified time and then imaged to visualize loss of NHR-25::GFP::AID*. Additional timepoints can be found in Supplementary Figure S2. Scale bars represent 5 µm. NHR-25::GFP::AID* degradation kinetics in (B) VPCs and (C) seam cells. Kinetics were determined by measuring NHR-25::GFP::AID* levels in L3s (as described above) exposed to 1 mM K-NAA or IAA. The graph depicts the mean normalized fluorescent intensity from 10 or more animals from a single experimental replicate. Error bars indicate standard deviation.

To further test the performance of TIR1::F2A::BFP::AID*::NLS for depleting an endogenous AID-tagged protein, we compared depletion in the eft-3p::TIR1::F2A::BFP::AID*::NLS; nhr-25::GFP::AID*::3xFLAG strain to depletion in the previously described eft-3p::TIR1::mRuby2; nhr-25::GFP::AID*::3xFLAG strain (Martinez *et al.* 2020). Similar to the AID*::GFP experiments, we performed single cell quantitative imaging experiments (Figure 5). With the *eft-3p::TIR1::mRuby2* transgene we observed undetectable NHR-25::GFP::AID*::3XFLAG in both seam cells and VPCs within 30 minutes of exposure to IAA or K-NAA (Figure 5 and Supplementary Figure S2). In contrast, we observed differences in IAA and K-NAA treatment using our eft-3p::TIR1::F2A::BFP::AID*::NLS; nhr-25::GFP::AID*::3xFLAG strain (Figure 5 and Supplementary Figure S2). K-NAA resulted in more rapid NHR-25::GFP::AID*::3xFLAG depletion by TIR1::F2A::BFP::AID*::NLS compared to IAA (Figure 5 and Supplementary Figure S2). In VPCs, NHR-25::GFP::AID* was robustly depleted after 60 minutes of exposure to K-NAA and undetectable after 90 minutes (Figure 5B and Supplementary Figure S2). There appeared to be tissue-specific differences in depletion rates as NHR-25::GFP::AID*::3xFLAG was undetectable in seam cells 60 minutes after exposure to K-NAA (Figure 5C and Supplementary Figure S2). Depletion rates were slower with IAA, taking an additional 30 minutes for TIR1::F2A::BFP::AID*::NLS to deplete NHR-25::GFP::AID*::3xFLAG to undetectable levels (Figure 5 and Supplementary Figure S2). These results suggest that: i) some element of our TIR1::F2A::BFP::AID*::NLS transgene is limiting depletion rate; ii) we reach the same depletion endpoint using our TIR1::F2A::BFP::AID*::NLS transgene; iii) there can be tissue-specific differences in depletion rates; and iv) K-NAA performed equal to or better than IAA in these depletion experiments. These results suggest that one should test IAA and K-NAA for depletion efficiency independently for each TIR1 expression system and AID*-tagged protein.

### eft-3p::TIR1::F2A::BFP::AID*::NLS produces comparable depletion phenotypes as *eft-3p::TIR1::mRuby2* for depleting both nuclear and cytoplasmic proteins

Given the slower depletion kinetics we observed in our eft-3p::TIR1::F2A::BFP::AID*::NLS strain for an AID*::GFP reporter and NHR-25::GFP::AID*::3xFLAG, we wanted to test the impact of this slowed depletion rate at the phenotypic level. We first tested the two TIR1 alleles on our nhr-25::GFP::AID*::3xFLAG strain (Table 2 and Supplementary Table S5). With the *TIR1::mRuby2* transgene and IAA, we observed that 61% of nhr-25::GFP::AID*::3xFLAG larvae arrested, and the remaining 39% of animals that made it to adulthood had gonadal abnormalities (Table 2 and Supplementary Table S5). With our TIR1::F2A::BFP::AID*::NLS transgene, we observed that 85% of larvae arrested, whereas 93% of animals that reached adulthood had gonadal abnormities (Table 2 and Supplementary Table S5). Surprisingly, with growth on K-NAA we did not observe any larval arrests for either strain, but all adult animals had gonadal defects (Table 2 and Supplementary Table S5). We next tested the two different TIR1 strains on an nhr-23::AID*::3xFLAG allele (Zhang *et al.* 2015). Similar to previous reports (Zhang *et al.* 2015), we observed a completely penetrant larval arrest with both the nhr-23::AID*::3xFLAG; eft-3p::TIR1::mRuby2 and nhr-23::AID*::3xFLAG, eft-3p::TIR1::F2A::BFP::AID*::NLS strains grown on either IAA or K-NAA. Interestingly, some of the arrested larvae produced by growth on K-NAA died (Supplementary Table S5), which we did not see on IAA and is consistent with a stronger phenotype. None of the strains tested presented any defects on MYOB or ethanol control plates (Table 2 and Supplementary Table S5). Finally, we wished to test an additional cytoplasmic AID*-tagged protein for depletion phenotypes. We chose DAF-15/Raptor, a lysosome-localized factor which causes larval arrest when depleted (Duong *et al.* 2020). Both *eft-3p::TIR1::mRuby2* and eft-3p::TIR1::F2A::BFP::AID*::NLS caused a completely penetrant larval arrest in animals carrying the daf-15::mNeonGreen::AID* (Table 3 and Supplementary Table S6). Strains grown on IAA arrested at L2, whereas strains grown on K-NAA arrested at L3 (Table 3 and Supplementary Table S6). Based on previous work (Duong *et al.* 2020), these results suggests that K-NAA is producing slower or less complete DAF-15::mNG::AID* depletion than IAA. For DAF-15::mNG::AID*, IAA appeared to perform better. For all strains tested, we observed wild-type growth on control media (Tables 2 and 3; Supplementary Tables S5 and S6). Together, these results indicated that our eft-3p::TIR1::F2A::BFP::AID*::NLS transgene performs comparably to the original *eft-3p::TIR1::mRuby2* with respect to phenocopying mutant phenotypes of NHR-23, NHR-25, and DAF-15.

**Table 2:** eft-3p::TIR1::F2A::BFP::AID*::NLS produces comparable depletion phenotypes to *eft-3p::TIR1::mRuby2* for depleting nuclear proteins

*eft-3p::TIR1* transgene	AID-tagged allele	Treatment	% WT developmental rate	% Larval arrest	% Gonadal abnormalities among adults
*TIR1::mRuby2*	n/a	Control	100	0	0
*TIR1::mRuby2*	n/a	IAA	100	0	0
*TIR1::mRuby2*	n/a	K-NAA	100	0	0
*TIR1::2A::BFP*	n/a	Control	100	0	0
*TIR1::2A::BFP*	n/a	IAA	100	0	0
*TIR1::2A::BFP*	n/a	K-NAA	100	0	0
*TIR1::mRuby2*	nhr-23(kry61[nhr-23::AID*::TEV-3xFLAG])	Control	100	0	0
*TIR1::mRuby2*	nhr-23(kry61[nhr-23::AID*::TEV-3xFLAG])	IAA	0	100	0
*TIR1::mRuby2*	nhr-23(kry61[nhr-23::AID*::TEV-3xFLAG])	K-NAA	0	100	0
*TIR1::2A::BFP*	nhr-23(kry61[nhr-23::AID*::TEV-3xFLAG])	Control	100	0	0
*TIR1::2A::BFP*	nhr-23(kry61[nhr-23::AID*::TEV-3xFLAG])	IAA	0	100	0
*TIR1::2A::BFP*	nhr-23(kry61[nhr-23::AID*::TEV-3xFLAG])	K-NAA	0	100	0
*TIR1::mRuby2*	nhr-25(wrd18[nhr-25::GFP^AID*:3xFLAG])	Control	100	0	0
*TIR1::mRuby2*	nhr-25(wrd18[nhr-25::GFP^AID*:3xFLAG])	IAA	39	61	100
*TIR1::mRuby2*	nhr-25(wrd18[nhr-25::GFP^AID*:3xFLAG])	K-NAA	100	0	100
*TIR1::2A::BFP*	nhr-25(wrd52[nhr-25::GFP^AID*:3xFLAG])	Control	100	0	0
*TIR1::2A::BFP*	nhr-25(wrd52[nhr-25::GFP^AID*:3xFLAG])	IAA	15	85	93
*TIR1::2A::BFP*	nhr-25(wrd52[nhr-25::GFP^AID*:3xFLAG])	K-NAA	100	0	100

Synchronized animals (*n* > 100) of the indicated genotype were grown on the indicated plates (control, IAA, K-NAA). These animals were scored for developmental rate (% animals that were L4 or adults following 48 h at 25°C), larval arrest prior to L4 (animals that failed to reach adulthood after 4 days), and gonadal defects. Data from *TIR1::2A::BFP* and TIR1::2A::BFP; nhr-23(kry61[nhr-23::AID*::TEV-3xFLAG]) represent two pooled experimental replicates. All other data are from a single experiment.

**Table 3:** eft-3p::TIR1::F2A::BFP::AID*::NLS produces comparable depletion phenotypes to *eft-3p::TIR1::mRuby2* for depleting cytoplasmic proteins

*eft-3p::TIR1* transgene	AID-tagged allele	Treatment	% WT developmental rate	% L2 arrest	% L3 arrest
*TIR1::mRuby2*	daf-15(re257[daf-15::mNG::AID*])	Control	100	0	0
*TIR1::mRuby2*	daf-15(re257[daf-15::mNG::AID*])	IAA	0	100	0
*TIR1::mRuby2*	daf-15(re257[daf-15::mNG::AID*])	K-NAA	0	0	100
*TIR1::2A::BFP*	daf-15(re257[daf-15::mNG::AID*])	Control	100	0	0
*TIR1::2A::BFP*	daf-15(re257[daf-15::mNG::AID*])	IAA	0	100	0
*TIR1::2A::BFP*	daf-15(re257[daf-15::mNG::AID*])	K-NAA	0	0	100

Synchronized animals (*n* = 120–240) of the indicated genotype were grown on the indicated plates (control, IAA, K-NAA). Animals were grown at 20°C for 4 days and scored for larval arrest. Wild-type (WT) developmental rate was determined by scoring animals that reached adulthood after 3 days at 20°C. L2 and L3 arrest were determined by animal size and gonadal development.

### A new suite of TIR1 driver strains compatible with red/green FP imaging

**Figure 6:**
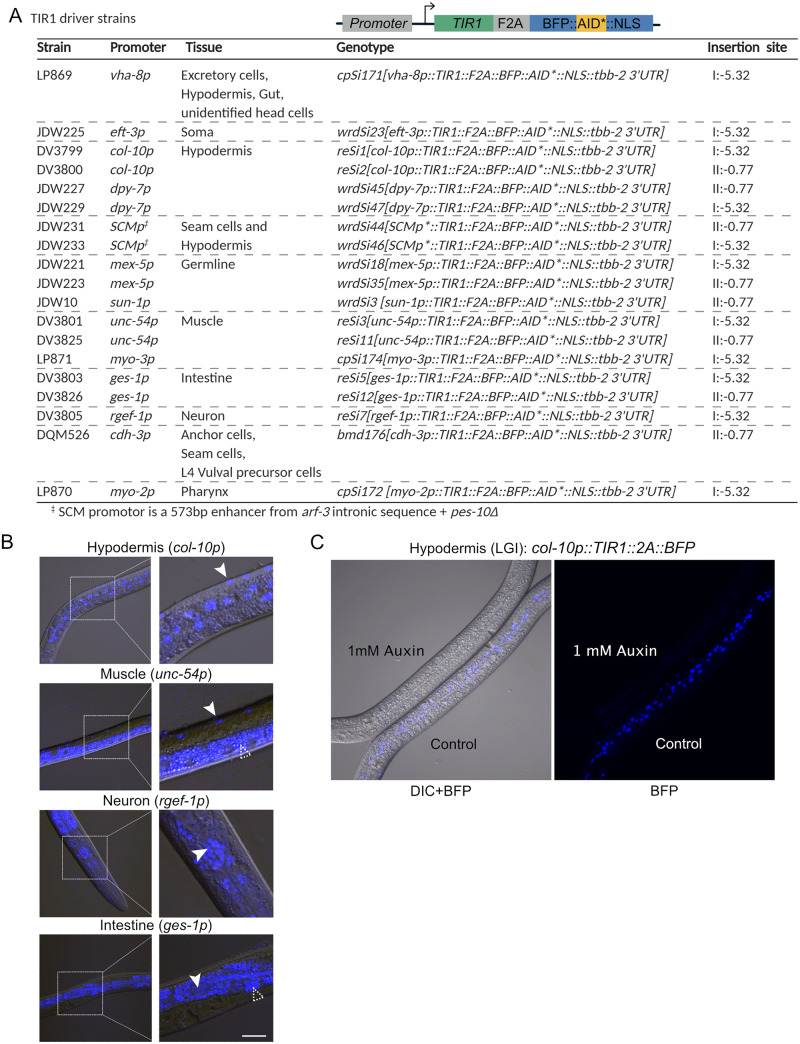
A new suite of TIR1 expression strains for tissue-specific depletion of AID-tagged proteins in *C. elegans*. (A) Table describing new suite of TIR1::F2A::BFP::AID*::NLS strains. Strain names, promoter driving TIR1, tissue of expression, genotype, and insertion site are provided for each strain. The insertion sites are the genomic loci where the MosI transposon landed in the *ttTi4348* and *ttTi5605* insertion alleles. We note that our knock-ins were generated using CRISPR/Cas9-mediated genome editing in wild-type animals or in strains stably expressing Cas9 in the germline; there is no MosI transposon in these loci in these genetic backgrounds. Created with BioRender.com. (B) BFP is detected in the expected nuclei of strains expressing TIR1 cassettes driven by *col-10p* (hypodermis), *unc-54p* (muscle), *ges-1p* (intestine), and *rgef-1p* (neurons). Representative BFP-expressing nuclei are indicated by solid arrows. Scale bars represent 20 µm. Note that the fluorescence signal at the bottom of the muscle image and surrounding the nuclei in the intestinal image is intestinal autofluorescence, indicated by an unfilled arrow with a dashed outline. (C) Functional test of TIR1 activity in a col-10p::TIR1::F2A::BFP::AID*::NLS strain (DV3799). Hypodermal BFP expression is lost when animals are exposed to 1 mM auxin for three hours, but not when similarly grown on control plates.

Having confirmed our TIR1::F2A::BFP::AID*::NLS transgene functioned effectively in both embryos and somatic cells, we wished to create a suite of strains for tissue-specific TIR1 expression (Figure 6A). We created chromosome I and II knock-ins expressing TIR1 in the germline (*mex-5p* and *sun-1p*), hypodermis (*dpy-7p* and *col-10p*), muscle *(unc-54p*), and intestine (*ges-1p)* (Figure 6 and Supplementary Figure S4). We also created chromosome I knock-ins expressing TIR1 in neurons (*rgef-1p*), pharynx (*myo-2p)*, body wall muscle (*myo-3p*), the anchor cell (*cdh-3*), as well as the excretory cell, hypodermis, and gut (*vha-8p*) (Figure 6 and Supplementary Figure S4). The *vha-8* promoter also drove expression in unidentified cells in the head. Additionally, we generated a strain expressing TIR1 in the hypodermal seam cells using a minimal SCMp enhancer (gift from Prof. Allison Woollard) with the *pes-10* minimal promoter (Figure 6A). Although we observed robust seam cell expression in this strain, we also detected hypodermal expression (unpublished data). We are making this strain available to the community but encourage careful evaluation before interpretation.

**Figure 7:**
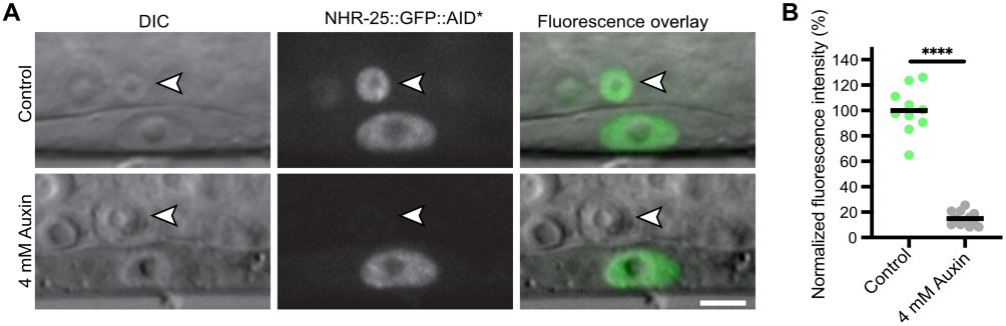
NHR-25::GFP::AID*::3xFLAG can be depleted in a cell-specific manner in a strain with undetectable TIR1 expression via a BFP reporter. (A) An anchor cell (AC)-specific TIR1 transgene (cdh-3p::TIR1::F2A::BFP::AID*::NLS) did not produce observable BFP in the AC. Crossing this strain to an nhr-25::GFP::AID*::3xFLAG allele resulted in depletion of NHR-25 in the AC when exposed to 4 mM auxin for 1 hr (indicated by white arrow with black outline). As expected, depletion of NHR-25 was not observed in the neighboring uterine cells or the underlying vulval precursor cells (VPCs). Scale bar represents 5 µm. (B) Quantification of NHR-25::GFP::AID*::3xFLAG in ACs following auxin (K-NAA) treatment. Individual data points from a single replicate with more than 10 animals per condition are presented. The horizontal black bar depicts the mean for each condition; **** indicates *P *<* *0.0001 by a two-tailed unpaired Student’s t-test. *P *<* *0.05 was considered statistically significant. Scale bars represent 5 µm.

All of the TIR1-expressing strains that we have deposited in the *Caenorhabditis* Genetics Center have detectable BFP expression that is lost when animals are shifted onto auxin plates, confirming TIR1 is active (Figures 2B and 6C; Supplementary Figures S2 and S4). However, an unanswered question is the importance of TIR1 expression levels for effective depletion of AID*-tagged proteins. Motivated by an interest in NHR-25 in gene regulation (Ward *et al.* 2013, 2014) and anchor cell (AC) invasion (Matus *et al.* 2015; Medwig and Matus 2017; Medwig-Kinney *et al.* 2020), we generated a strain to study early events in AC differentiation (DQM623; Figure 7). The *cdh-3* promoter drives transgene expression in the AC but not vulval precursor cells (VPCs) during early AC differentiation (Matus *et al.* 2015). The promoter becomes active in L4 VPCs and also drives expression in seam cells and neurons (Matus *et al.* 2015). We generated a cdh-3p::TIR1::F2A::BFP::AID*::NLS strain, but were not able to detect any BFP expression. To perform a functional test, we crossed the nhr-25::GFP::AID*::3xFLAG allele into the strain harboring cdh-3p::TIR1::F2A::BFP::AID*::NLS. We had previously shown significant depletion of NHR-25 in ACs and VPCs using a strongly expressed *eft-3p::TIR1::mRuby2* transgene (Martinez *et al.* 2020). Strikingly, we observed auxin-dependent depletion of NHR-25::GFP::AID*::3xFLAG in the AC but no depletion in the adjacent VPCs (Figure 7). Thus, even if the presence of TIR1 is undetectable through BFP reporter expression, there may still be a sufficient amount of TIR1 to deplete proteins of interest.

### Vectors to generate FP::AID* knock-ins

There are two commonly used CRISPR/Cas9 editing strategies currently used in *C. elegans* (Dickinson and Goldstein 2016; Nance and Frøkjær-Jensen 2019)*.* One uses injection of Cas9 ribonucleoprotein complexes and typically provides linear DNA repair templates (Paix *et al.* 2015; Dokshin *et al.* 2018; Ghanta and Mello 2020). The other approach uses injection of plasmids expressing Cas9 and sgRNA, and typically provides repair templates as plasmids with selectable markers (Dickinson *et al.* 2013, 2015; Norris *et al.* 2015; Schwartz and Jorgensen 2016). We created plasmids to facilitate assembly of repair templates for both of these genome editing strategies.

**Figure 8:**
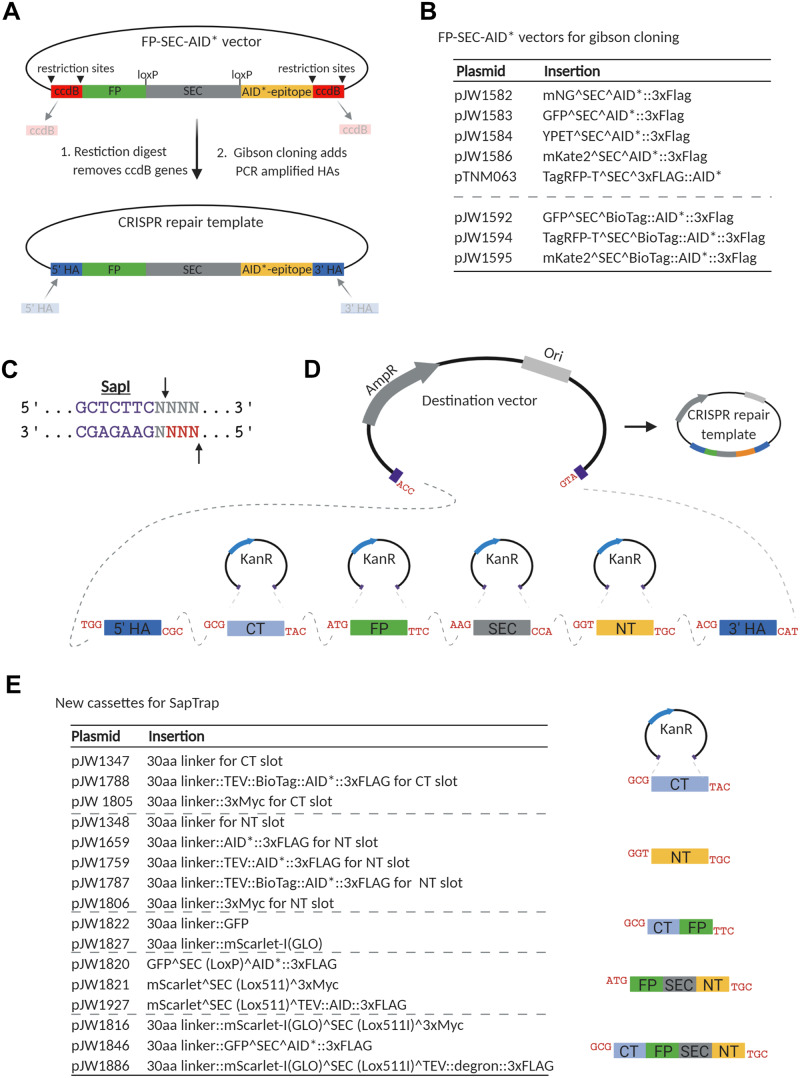
A collection of vectors to generate FP::AID* knock-ins through Gibson cloning and a suite of new vectors for the SapTrap cloning system. (A) Schematic of the AID*-containing vectors produced by modifying the set of vectors originally described by Dickinson *et al.* (2015). An AID* epitope was inserted downstream of the loxP-flanked SEC. New repair templates for CRISPR/Cas9-mediated genome editing can be produced by restriction digestion of the vector and Gibson cloning of PCR-derived 5’ and 3’ homology arms (5’HA and 3’HA), as described (Dickinson *et al.* 2015). Counter-selection against the parent vector is provided by ccdB cassettes. Created with BioRender.com. (B) A suite of new FP::AID* SEC plasmids. The vectors described in Dickinson *et al.* (2015) have been modified to insert an AID* or 23 amino acid biotin acceptor peptide (BioTag)::AID* cassette between the SEC and 3xFLAG cassette. (C) SapI is a type II restriction enzyme that cuts one base pair and four base pairs outside of its binding site, allowing for the generation of programable 3 bp sticky ends. D) SapTrap cloning facilitates single-reaction cloning of multiple fragments, in the correct order, into a single repair template plasmid. Specific sticky ends are used for specific cassettes as described by Schwartz *et al.* (2016). Created with BioRender.com. E) Table of new vectors generated for the SapTrap CT and NT slots. Our initial assembly efficiencies were sub-optimal, and we found that reducing the number of fragments assembled improved our efficiencies. We have generated a set of multi-cassettes where partial assemblies (CT-FP, FP-SEC-NT, and CT-FP-SEC-NT) have been cloned, simplifying the SapTrap reactions and reducing the number of fragments required. 5’HA, 5’ homology arm; 3’HA, 3’ homology arm; FP, fluorescent protein; SEC, self-excising cassette; CT, C-terminal connector; NT, N-terminal connector.

First, we generated a set of vectors to create SEC-selectable plasmid repair templates. We took a set of vectors which use Gibson assembly to generate the final repair template (Dickinson *et al.* 2015) and introduced AID* sequences upstream of the 3xFLAG epitope. This set of vectors allows for tagging genes with mNeonGreen, GFP, YPET, mKate2, and TagRFP-T along with AID*::3xFLAG epitopes (Figure 8 and Supplementary Table S2). Methods in *C. elegans* using biotin ligases and biotin acceptor peptides have recently been described for protein affinity purification (Waaijers *et al.* 2016), proximity labeling (Branon *et al.* 2018), native chromatin purification (Ooi *et al.* 2010), and cell-type specific nuclei purification (Steiner *et al.* 2012). To support these approaches, we have made a set of FP^SEC^BioTag::AID*::3xFLAG vectors with GFP, TagRFP-T, and mKate2 (Figure 8 and Supplementary Table S2).

Another approach to generate plasmid repair templates with detectable markers is a Golden Gate assembly approach known as SapTrap (Engler *et al.* 2009; Schwartz and Jorgensen 2016). A library of SapTrap donor plasmids contain protein tags, selectable markers, and fluorescent proteins; these tags can be assembled in a desired order in a one-tube isothermal reaction to allow the creation of new repair templates (Schwartz and Jorgensen 2016; Dickinson *et al.* 2018) (Figure 8D). When trying to create SapTrap assemblies containing nine fragments, we encountered very poor cloning efficiencies. We therefore created a series of pre-assembled “multi-cassettes,” where we combined fragments that we frequently use (Figure 8E and Supplementary Table S2). These constructs contain inserts for SapTrap N-terminal and C-terminal connector modules, as described by Schwartz et al. (2016) and include various combinations of AID* cassettes, and epitopes for protein purification or detection (3xMyc, 3xFLAG, BioTag). This approach restored high cloning efficiency. For our most commonly used vectors, we have generated constructs containing full assemblies of the knock-in epitope, lacking only the homology arms. PCR amplifying homology arms with SapI sites and appropriate connectors allows high-efficiency generation of repair templates.

**Figure 9:**
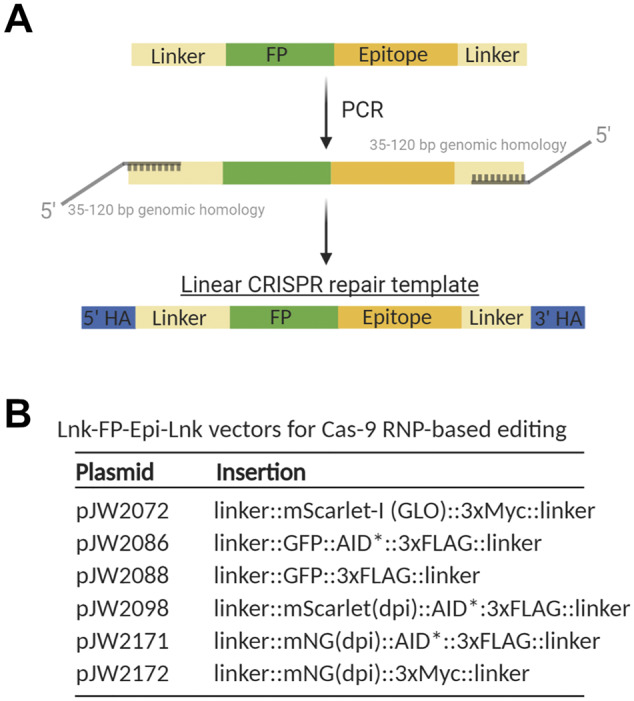
A collection of vectors to generate FP::AID* knock-ins using linear repair templates. (A) Schematic primer design to generate linear repair templates by PCR. Primers with homology to the cassette and 5’ homology to the desired integration site are used to amplify a dsDNA repair template. 35-120 bp homology arms (HA in figure) are recommended, as previously described (Paix *et al.* 2014; 2015; Dokshin *et al.* 2018). Created with BioRender.com. (B) A set of vectors to generate repair templates for Cas9 ribonucleoprotein complex (RNP)-based genome editing. FP (fluorescent protein) and FP::AID* cassettes are flanked by flexible linker sequences. A 30 amino acid sequence is at the 5’ end of the cassette, and a 10 amino acid sequence is at the 3’ end of the cassette. This design provides flexibility for designing repair templates for N-terminal, C-terminal, or internal tagging. GLO=Germline optimized using algorithm (Fielmich *et al.* 2018); dpi=silent mutations to remove piRNA binding sites (dpi) to promote germline expression (Zhang *et al.* 2018).

Using Cas9 ribonucleoprotein complexes with linear repair templates is a common cloning-free approach in *C. elegans* (Paix *et al.* 2014; 2015), and recent refinements have further boosted editing efficiency (Dokshin *et al.* 2018; Ghanta and Mello 2020). We have gave generated repair templates to tag proteins with GFP, germline optimized mNeonGreen, and germline optimized mScarlet-I (Fielmich *et al.* 2018; Zhang *et al.* 2018) (Figure 9B and Supplementary Table S2). All plasmids include a Myc or FLAG epitope for antibody-based detection, and we have versions with or without an AID* cassette. These vectors are suitable templates for PCR amplification to generate linear repair templates with short homology arms. FPs can be easily exchanged to generate new constructs by PCR linearization and Gibson cloning. Linkers flanking the FP allow flexibility in targeting genes of interest and reduce functional interference, permitting N-terminal, C-terminal, or internal tagging.

## Discussion

The original *C. elegans* AID system employed a TIR1::mRuby2 transgene, which was useful for visualizing TIR1 expression and cellular localization (Zhang *et al.* 2015). However, for applications where red fluorescent protein imaging is desired, the mRuby2 expression could increase background and hamper imaging analysis. We therefore developed a complementary construct containing a TIR1::F2A::BFP::AID*::NLS transgene (Figure 2A). TIR1 is unlabeled, and nuclear-localized BFP provides a readout for TIR1 expression (Figures 2, 3, and 6; Supplementary Figure S2). AID*-tagged BFP is degraded in the presence of auxin, confirming TIR1 activity via degradation of an internal control (Figures 2 and 3, and Supplementary Figure S2). Recent work from our lab shows that our new TIR1 strains are effective for experiments where red FP imaging is desired (Ragle *et al.* 2020).

Our eft-3p::TIR1::F2A::BFP::AID*::NLS transgenes produced the same degree of depletion of AID-tagged substrates compared to an *eft-3p::TIR1::mRuby2* transgene (Zhang *et al.* 2015), but the depletion kinetics were slower (Figures 4 and 5). Initially concerned about the impact of this slower degradation rate, we tested whether it impacted the phenotypes observed following depletion of two AID*-tagged nuclear proteins (NHR-23 and NHR-25) and an AID*-tagged cytoplasmic protein (DAF-15). In all cases we observed comparable penetrance of expected phenotypes (Tables 2 and 3). Additionally, our sun-1p::TIR1::F2A::BFP::AID*::NLS transgene produced equivalent mNG::AID*::PAR-3 depletion and resultant synchronous cell division as compared to a *sun-1p::TIR1::mRuby2* transgene (Figure 2, C and D). Similarly, using a mex-5p::TIR1::F2A::BFP::AID*::NLS transgene we observed complete sterility following NHR-23 or SPE-44 depletion, identical to that observed using *pie-1p* or *sun-1p::TIR1::mRuby2* transgenes (Kasimatis *et al.* 2018; Ragle *et al.* 2020). However, there may be cases where depletion rates affect phenotypic preference, so this point should be taken into consideration when choosing which TIR1 transgene to use.

One open question is why did our BFP reporter slow the depletion of other AID*-tagged substrates? Inefficient F2A processing could lead to a TIR1::2A::BFP fusion protein that could be degraded. However, we were unable to detect such a fusion protein by western blotting, arguing against this possibility (Figure 3B). Another explanation is that some component of the system is limiting under these conditions. Possible candidates for the limiting factor include TIR1 itself, the SCF ligase with which TIR1 interacts (Martinez *et al.* 2020), or proteasomal activity. Pan-neuronal TIR1 expressed from an extrachromosomal array produced much stronger depletion of an AID*-tagged substrate compared to the equivalent TIR1 construct in single-copy (O. Hobert, personal communication). Although this observation needs to be explored further, it supports the idea that TIR1 is limiting in some cases. Strong expression of both AID*::GFP and BFP::AID*::NLS could sequester the SCF ligase or proteasome if they were limiting. This sequestration might be expected to produce documented reduction-of-function phenotypes such as defects in cell fate and differentiation, meiotic defects, or embryonic lethality (Kipreos *et al.* 1996; Nayak *et al.* 2002; Kamath *et al.* 2003). As we did not observe any such defects, we favor a model where TIR1 is limiting in our system. Going forward, it will be important to test this model by boosting TIR1 expression using tools such as site-specific integration of arrays or recently described bipartite gene expression systems (Yoshina *et al.* 2016; Nonet 2020). Another approach may be to fuse the Skp1 subunit of the SCF ubiquitin ligase to TIR1, which has resulted in enhanced degradation efficiency of AID-tagged proteins (Kanke *et al.* 2011). Determining which components of the system are limiting and boosting TIR1 activity has the potential to address the reported issue of incomplete degradation of some AID-tagged proteins and a failure to obtain null phenotypes (Patel and Hobert 2017; Serrano-Saiz *et al.* 2018; Duong *et al.* 2020)

We have compared the performance of natural auxin (IAA) with a synthetic, water-soluble analog (K-NAA) using quantitative imaging and phenotypic assays. In many cases, they produced identical results. For NHR-25::GFP::AID*::3xFLAG depletion, we observed more rapid depletion with K-NAA in two cell types using our eft-3p:: TIR1::F2A::BFP::AID*::NLS (Figure 5). However, interestingly K-NAA did not produce the larval arrest phenotype in these animals, whereas IAA did (Table 2). It is unclear what is behind the discrepancy between these results; we will need to determine in which cells NHR-25 is acting to promote larval development and how IAA and K-NAA affect its depletion in this cell type. Additionally, daf-15::mNG::AID*animals grown on IAA arrested at an earlier larval stage than animals treated with K-NAA (Table 3) consistent with faster and/or more robust depletion (Duong *et al.* 2020). Our results do not indicate that one form of auxin is consistently superior, but rather that one needs to empirically test each auxin, depending on the AID-tagged gene, the TIR1 transgene, and the tissue in which depletion is occurring. As the community tests IAA and K-NAA on more AID*-tagged factors, it will be important to look for patterns that might emerge in order to better predict which auxin will perform better in specific assays.

For many applications, the AID system offers a powerful method to conditionally degrade proteins in specific tissues and at specific points in development. However, as the system has gained popularity, particular challenges have emerged. Although they do not dampen our enthusiasm for the AID system, it is important to be aware of them. The first is the previously discussed issue of incomplete degradation. The second issue is that auxin-independent, TIR1-dependent degradation of certain AID-tagged proteins has been documented in both human cells and in *C. elegans* (Zasadzińska *et al.* 2018; Sathyan *et al.* 2019; Martinez *et al.* 2020; Schiksnis *et al.* 2020). Although there are recent solutions to this issue, such as alternate AID systems (Li *et al.* 2019) or additional regulatory components that block auxin-independent degradation of AID-tagged proteins (Sathyan *et al.* 2019), these methods are not compatible with existing *C. elegans* strains using minimal AID tags. Engineering an improved TIR1 that does not promote auxin-independent degradation of minimal AID-tagged proteins would be the preferable solution. A strong candidate are recently described TIR1 mutations that produce 670-1000x stronger binding to a modified auxin, reducing the amount of auxin required for target knockdown (Nishimura *et al.* 2020; Yesbolatova *et al.* 2020). This reagent would be compatible with and improve the performance of the collection of minimal AID-tagged strains, which the *C. elegans* community has already generated. Finally, AID-tagging in rare cases can disrupt protein function. For example, an mNeonGreen::AID* tag caused a mild hypomorph of *unc-3* in the absence of TIR1 and auxin, suggesting that the presence of the AID* tag was interfering with protein levels (Patel and Hobert 2017). More examples are required to determine rules for optimal AID tag placement in both structured and unstructured domains of proteins. As a precaution, we tend to use long 10-30 amino acid flexible linker sequences to space the AID* tag away from the protein of interest.

The ability to rapidly deplete proteins with temporal and cellular resolution allows precise dissection of the roles of gene products in developmental processes of interest. With the ever-increasing efficiency of genome editing and continued refinement of the AID system, one can envision creating libraries of FP::AID*-tagged genes covering the genome and a bank of TIR1 strains to allow depletion in virtually all cell types. Together, this collection of strains and vectors should facilitate efficient generation and depletion of new FP::AID*-tagged proteins.
